# Fokker–Planck and Fortet Equation-Based Parameter Estimation for a Leaky Integrate-and-Fire Model with Sinusoidal and Stochastic Forcing

**DOI:** 10.1186/2190-8567-4-4

**Published:** 2014-04-17

**Authors:** Alexandre Iolov, Susanne Ditlevsen, André Longtin

**Affiliations:** 1 Department of Mathematics and Statistics, University of Ottawa, Ottawa, Canada; 2 Department of Mathematical Sciences, University of Copenhagen, Copenhagen, Denmark; 3 Department of Physics and Center for Neural Dynamics, University of Ottawa, Ottawa, Canada

**Keywords:** First-passage times, Stochastic neuron models, Parameter estimation from stopping times, Fortet integral equation, Fokker–Planck equation

## Abstract

**Abstract:**

Analysis of sinusoidal noisy leaky integrate-and-fire models and comparison with experimental data are important to understand the neural code and neural synchronization and rhythms. In this paper, we propose two methods to estimate input parameters using interspike interval data only. One is based on numerical solutions of the Fokker–Planck equation, and the other is based on an integral equation, which is fulfilled by the interspike interval probability density. This generalizes previous methods tailored to stationary data to the case of time-dependent input. The main contribution is a binning method to circumvent the problems of nonstationarity, and an easy-to-implement initializer for the numerical procedures. The methods are compared on simulated data.

**List of Abbreviations:**

LIF: Leaky integrate-and-fire

ISI: Interspike interval

SDE: Stochastic differential equation

PDE: Partial differential equation

## 1 Introduction

Information processing in the nervous system is carried out by spike timings in neurons. To study the neural code in such a complicated system, a first step is to understand signal processing and transmission in single neurons. Stochastic leaky integrate-and-fire (LIF) neuronal models are a good compromise between biophysical realism and mathematical tractability, and are commonly applied as theoretical tools to study properties of real neuronal systems. A central issue is then to perform statistical inference from experimental data and estimate model parameters. Many electrophysiological experiments on neurons, namely extra-cellular recordings, are only capable of detecting the time of the spike and not the detailed voltage trajectory leading up to the spike. Estimating the parameters of the LIF model from this type of data is equivalent to estimating the parameters of a stochastic model from the statistics of the first-passage times only. A common assumption is that the data are well described by a renewal process, thus basing the statistical inference on the interspike intervals (ISIs), assuming these are realizations of independent and identically distributed random variables. Since only partial information about the process is available, the statistical problem becomes more difficult, and no explicit expression for the likelihood is available.

Different methods have been proposed. In the seminal paper [1], a point process approach is proposed. The spike trains of a collection of neurons are represented as counting processes. Time is discretized and the point processes approximated by 0–1 time series. Then the probability of firing in the next time interval is modeled as a function of the spike history. In this way, maximum likelihood estimation is feasible. External stimuli are not considered. In [2], a numerically involved moment method is developed. It uses the first two moments of the first-passage times of the Ornstein–Uhlenbeck process to a constant threshold, which are given as series expressions, and equates them to their empirical counterparts. In [3,4], certain explicit moment relations derived from the Laplace transform of the first-passage time distribution are applied, but these are only valid under stimulation (supra-threshold regime). In [5], inference is based on numerical inversion of the Laplace transform. In [6], a functional of a three-dimensional Bessel bridge is applied to obtain a maximum likelihood estimator. None of these methods are feasible to extend to the time-inhomogeneous case, which is of our interest. In [7,8], an integral equation is used to derive an estimator in the time-homogeneous setting. This approach is readily extended to time varying input, which we will explore in this paper. Some of the above methods are compared in [9]. Finally, a review of estimation methods is provided in [10]. 

Many sensory stimuli, like sound, contain an oscillatory component [11,12]. Such inputs will cause oscillating membrane potentials in the neuron, generating rhythmic spiking patterns. The oscillation frequency determines the basic rhythm of spiking, and is considered to be significant for neuronal information processing. The dynamics of periodically forced neuron models have been extensively studied; see [13-18] and references therein. Even so, attempts to solve the estimation problem in these nonstationary settings have been rare. One problem is that the ISIs are no longer independent nor identically distributed. In [19], a more complicated model with linear filters is considered, allowing also for the spike history to influence the membrane potential dynamics. The estimation problem is solved through numerical solutions to the Fokker–Planck equation, and it is shown that the log-likelihood is concave, thus ensuring a global maximum; see also [20,21]. Because their model is more involved, some approximations to the solution of the Fokker–Planck equation are applied, to ensure acceptable computing times. We will apply the full Fokker–Planck equation to solve our estimation problem, since the computing time is always lower than 2 seconds for a sample size of 1000 spikes. 

In this paper, we thus describe and discuss two methods to estimate parameters of LIF models with the added complexity of a time-varying input current. We assume that the time-varying current is a sinusoidal wave, but we believe that the approaches generalize to an arbitrary periodic forcing with known frequency. One approach relies on the Fortet integral equation, which is readily extended to the time-inhomogeneous case. An advantage of this approach is that if the transition density of the diffusion in the LIF model is known, as is the case for the Ornstein–Uhlenbeck and the Feller model, the computational burden is limited. A second approach involves numerical solution of the Fokker–Planck equation, where the time-dependence is explicitly accounted for. After a numerical differentiation, the likelihood function can be calculated providing the maximum likelihood estimator. Nevertheless, we chose an alternative loss function which seems marginally more robust, directly comparing the survival function provided by the solution of the Fokker–Planck equation with its empirical counterpart. The two approaches give similar results and they are more carefully compared in the supplementary online material. Both methods need sensible starting values for the optimization algorithms, and we provide an easy-to-implement initializer. The estimation procedures are compared on simulated data and we find that both algorithms are able to find estimates close to the true values for several different dynamical regimes. We find that for small sample sizes the Fokker–Planck algorithm can be considered marginally preferable, whereas for larger sample sizes the Fortet algorithm becomes marginally superior. Moreover, at high frequencies of the sinusoidal forcing, the Fortet is better at identifying the parameters, though in general there is less information in the data to distinguish between a constant input and the amplitude of the periodic forcing.

## 2 Model

The time evolution of the voltage of a spiking neuron is modelled by a stochastic process, *V*, given as solution to the following stochastic differential equation (SDE):

(1) $$\begin{array}{rl} & dV(t)=\left(\mu -\frac{V(t)}{\tau }+A\sin(\omega t)\right)dt+\sigma dW(t),\\ & t_{0}=0;\;V(t_{0})=v_{0},\\ & t_{n}=\inf\left\{t>t_{n-1}:V(t)=v_{\mathrm{th}}\right\}\;\text{for }n\geq 1,\\ & \left\{ \begin{array}{c} V(t_{n}^{+})=v_{0},\\ J_{n}=t_{n}-t_{n-1}. \end{array}\right. \end{array}$$

Here, *μ* is a bias current acting on the cell, *τ* is the decay time, *A* and *ω* are the amplitude and (angular) frequency of the sinusoidal current acting on the cell, *σ* is the strength of the stochastic fluctuations, $W={\left\{W_{t}\right\}}_{t\geq 0}$ is a standard Wiener process, and $t_{n}^{+}$ denotes the right limit taken at $t_{n}$. A spike occurs when the membrane voltage V(t) crosses a voltage threshold, $v_{\mathrm{th}}$, and then V(t) is instantaneously reset to the resting potential $v_{0}$. The difference between subsequent spike times, $J_{n}=t_{n}-t_{n-1}$, is called the interspike interval (ISI).

We will assume that *τ* is known (but see Sect. 6 for a discussion of the alternative) and nondimensionalize Eq. (1) as follows:

$$\begin{array}{rcl} s & = & \frac{t}{\tau },\;X_{s}=\frac{V(t)-v_{0}}{v_{\mathrm{th}}-v_{0}},\;W_{s}=\frac{W(t)}{\sqrt{\tau }},\;x_{\mathrm{th}}=1,\\ \alpha & = & \frac{\mu \tau }{v_{\mathrm{th}}-v_{0}},\;\beta =\frac{\sigma \sqrt{\tau }}{v_{\mathrm{th}}-v_{0}},\;\gamma =\frac{A\tau }{v_{\mathrm{th}}-v_{0}},\;\Omega =\omega \tau \end{array}$$

to obtain

(2) $$\begin{array}{rl} & dX_{s}=\left(\alpha -X_{s}+\gamma \sin(\Omega s)\right)ds+\beta dW_{s},\\ & s_{0}=0;\;X_{s_{0}}=0,\\ & s_{n}=\inf\{s>s_{n-1}:X_{s}=x_{\mathrm{th}}=1\}\;\text{for }n\geq 1,\\ & \left\{ \begin{array}{c} X_{s_{n}^{+}}=0,\\ I_{n}=s_{n}-s_{n-1}, \end{array}\right. \end{array}$$

where we have defined $I_{n}=J_{n}/\tau$. We can also write the dynamics between two spike times $s_{n}$ and $s_{n+1}$ in terms of elapsed time since the last spike, $s^{\prime }=s-s_{n}$, $s^{\prime }<I_{n+1}$,

(3) $$\begin{array}{rl} & dX_{s^{\prime }}=\left(\alpha -X_{s^{\prime }}+\gamma \sin\left(\Omega \left(s^{\prime }+\phi_{n}\right)\right)\right)ds^{\prime }+\beta dW_{s^{\prime }},\\ & \left\{ \begin{array}{c} s^{\prime }=s-s_{n},\\ \phi_{n}=s_{n}\mathrm{mod}\frac{2\pi }{\Omega }. \end{array}\right. \end{array}$$

This form of the dynamics highlights that this is not a renewal process since different trajectories between spikes have different phase shifts $\phi_{n}=s_{n}$ modulo 2π/Ω. This will be important in the following discussion. The shape of the ISI distribution depends on the model parameters, and it is natural to divide the parameter space in different regimes characterized by their qualitative behavior. Four distinct parameter regimes will be considered; supra-threshold, critical, subthreshold, and supersinusoidal. To understand the reasoning behind the regime names, observe that in the absence of noise, β=0, the deterministic model will produce spikes if and only if

$$\alpha +\frac{\gamma }{\sqrt{1+\Omega^{2}}}>1,$$

see the discussion in [14], which can be directly inferred from the solution in Eq. (12) below. In both the supra-threshold and supersinusoidal regimes, $\alpha +\gamma /\sqrt{1+\Omega^{2}}>1$. The difference between the two is that in the supra-threshold regime the constant bias current alone is sufficient for spikes to occur, also in absence of noise, that is, α>1. In the supersinusoidal regime, the sinusoidal current is necessary for spikes to occur in absence of noise, that is, $\alpha +\gamma /\sqrt{1+\Omega^{2}}>1$ and α≤1. In the critical regime, the sum of the two terms is just barely enough to guarantee deterministic spiking, that is $\alpha +\gamma /\sqrt{1+\Omega^{2}}\approx 1$. Finally, in the subthreshold regime, there would be no spikes without the noise, $\alpha +\gamma /\sqrt{1+\Omega^{2}}<1$.

**Fig. 1:**
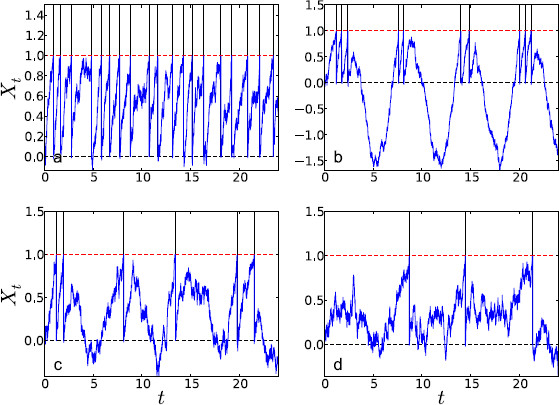
Example trajectories from Eq. (2) for the four different parameter regimes using the parameter values given in Table 1. **a** supra-threshold, **b** supersinusoidal, **c** critical, **d** subthreshold. In the supra-threshold regime spikes occur regularly and often; in the supersinusoidal regime spikes cluster near the peak of the sine wave; in the critical regime they occur less often; and in the subthreshold regime, spikes occur rarely. For all regimes, Ω=1

**Fig. 2:**
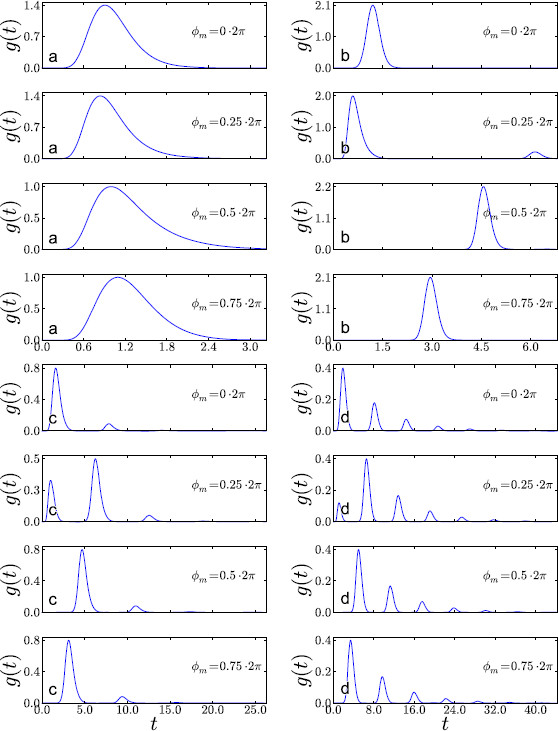
The four different parameter regimes using the parameter values given in Table 1. Illustrated are the probability density functions, $g_{\phi_{m}}(t)$, for representative $\phi_{m}=2\pi /\Omega \times \{0,0.25,0.5,0.75\}$. Varying $\phi_{m}$ has, for the most part, the effect of shifting the curves laterally, while varying *α*, *β*, *γ* changes their characteristic form. For all regimes, Ω=1. **a** supra-threshold, **b** supersinusoidal, **c** critical, **d** subthreshold

**Fig. 3:**
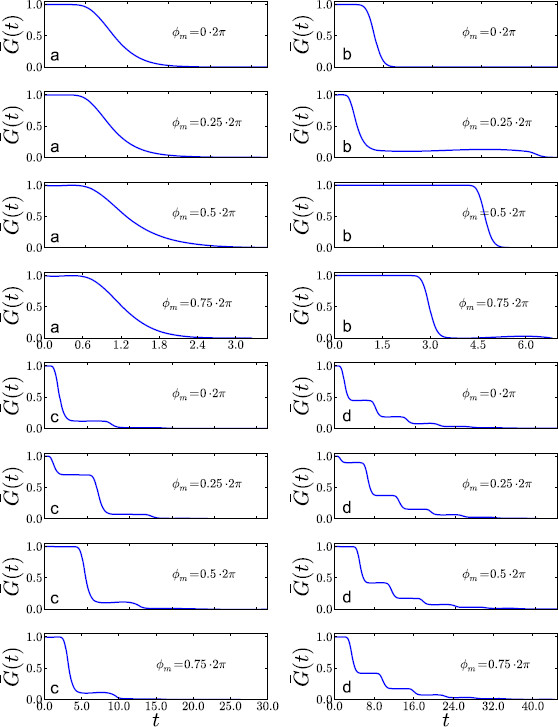
The four different parameter regimes using the parameter values given in Table 1. Illustrated are the survivor distribution functions, ${\bar{G}}_{\phi_{m}}(t)$, for representative $\phi_{m}=2\pi /\Omega \times \{0,0.25,0.5,0.75\}$. Varying $\phi_{m}$ has, for the most part, the effect of shifting the curves laterally, while varying *α*, *β*, *γ* changes their characteristic form. **a** supra-threshold, **b** supersinusoidal, **c** critical, **d** subthreshold

Table 1 tabulates examples of corresponding parameter values for each regime, while Fig. 1 shows examples of individual voltage trajectories and their associated spike trains. Figures 2 and 3 illustrate how each regime behaves for selected *ϕ*’s by plotting the survivor distribution, ${\bar{G}}_{\phi }(t)$, and the probability density, $g_{\phi }(t)$, both defined in Eq. (4) below. 

**Table 1:** Example of *α*, *β*, *γ* parameter values for the different regimes, given Ω=1

Regime name	*α*	*β*	*γ*
Supra-threshold	1.40	0.30	0.14
Supersinusoidal	0.10	0.30	1.98
Critical	0.50	0.30	0.71
Subthreshold	0.40	0.30	0.57

With regards to Figs. 2 and 3, it is worth noting explicitly that combinations of noise and sinusoidal forcing can cause firing patterns in which spikes are phase locked, but skip a certain number of cycles. This leads to multimodal ISI densities. There are many different dynamical mechanisms that can yield such patterns, and the particular correlations between the ISIs will depend on the underlying voltage dynamics (which, in our case, we assume to be given by Eq. (1)); in particular, it may be difficult to distinguish whether the dynamics are subthreshold or supra-threshold, since both can show similar ISI densities; see [22]. 

### 2.1 Basic ISI Probability Density Functions

Here, we introduce the notation for the probability density, distribution and survival functions of $I_{n}$, an ISI arising from a trajectory produced by Eq. (3),

(4) $$\begin{array}{rl} g_{\phi }(\tau )d\tau & :=P\left(I_{n+1}\in [\tau ,\tau +d\tau )|\phi_{n}=\phi \right)\;\text{(probability density)},\\ G_{\phi }(t) & :=P(I_{n+1}\leq t|\phi_{n}=\phi )=\int_{0}^{t}g_{\phi }(\tau )d\tau \;\text{(cumulative distribution)},\\ {\bar{G}}_{\phi }(t) & :=P(I_{n+1}>t|\phi_{n}=\phi )=1-G_{\phi }(t)\;\text{(survivor distribution)}. \end{array}$$

The subscript *ϕ* is to stress that *g*, *G*, and $\bar{G}$ depend on the value of $\phi_{n}$ in Eq. (3). This is the formal statement that in a sinusoidally-driven neuron, the interspike intervals are not identically distributed, and are only independent conditioned on the sinusoidal phase at an interval’s onset. Knowing these distributions would provide the likelihood function, offering estimation by the preferred method of choice, the maximum likelihood estimator. Unfortunately, explicit expressions for the ISI distribution are not available except for the special case of γ=0 and α=1; see [3]. Different representations of the likelihood function are available though, see [23], one of which we will use below. 

### 2.2 Fokker–Planck Equation with Absorbing Boundaries

The Fokker–Planck equation is a partial differential equation (PDE) describing the evolution of the probability density, f(x,t), of $X_{t}$. For the sinusoidally-forced Ornstein–Uhlenbeck process, Eq. (3), with the threshold $x_{\mathrm{th}}=1$, the PDE is

(5) $$\begin{array}{rl} \partial_{t}f^{\left(\phi \right)}(x,t)= & -\partial_{x}\left[\left(\alpha -x+\gamma \sin\left(\Omega (t+\phi )\right)\right)\cdot f^{\left(\phi \right)}\right]\\ & +\partial_{x}^{2}\left[\frac{\beta^{2}}{2}f^{\left(\phi \right)}\right],\;x\in (-\infty ,1). \end{array}$$

Due to the reset, we have that at time t=0, $X_{t}=0$ and so for the initial conditions we can write

(6) $$f^{\left(\phi \right)}(x,t=0)=\delta (x),$$

where δ(⋅) is the Dirac delta function. The spike is represented as a zero boundary condition for *f* at x=1

f(1,t)=0.

The natural way of using the Fokker–Planck equation in first-hitting-times problems is as follows. Denote the integral of $f^{\left(\phi \right)}$ by $F^{\left(\phi \right)}(x,t)=\int_{\xi \leq x}f^{\left(\phi \right)}(\xi ,t)d\xi$. $F^{\left(\phi \right)}(x,t)$ can be related to the ISI’s survivor distribution function, ${\bar{G}}_{\phi }(t)$, by

(7) $${\bar{G}}_{\phi }(t)=F^{\left(\phi \right)}(1,t).$$

Equation (7) forms the basis of one of the methods below for estimating the structural parameters from the observed data.

Since Eq. (5) has to be solved numerically, we will need to truncate its domain from below. The most natural way to do this, given the dynamics, is to impose reflecting boundary conditions at some $x=x_{-}\ll (\alpha -\gamma /\sqrt{1+\Omega^{2}})$ where the probability mass is very small. For the left (lower) limit of the computational domain, we use the formula

$$x_{-}=\min\left(\underset{\mathrm{mean}}{\underset{︸}{\alpha -\gamma /\sqrt{1+\Omega^{2}}}}-2\underset{std.\;dev.}{\underset{︸}{\beta /\sqrt{2}}},-0.25\right).$$

This choice requires some explanation. In the t→∞ limit, the distribution of $X_{t}$ in Eq. (3) *without* thresholding is Gaussian with mean given by Eq. (12) (below) and variance equal to $\beta^{2}/2$. Thus, to truncate the computational domain for the thresholded process from below, we take the lowest value of the asymptotic mean, $\alpha -\gamma /\sqrt{1+\Omega^{2}}$, then from this we subtract two standard deviations, $2\beta /\sqrt{2}$ and set the result to be the lower bound, $x_{-}$. Finally, if this value for $x_{-}$ happens to be larger than −0.25, we enforce that $x_{-}\leq -0.25$.

Numerical considerations lead us to solve for *F*, instead of *f*, since delta functions are difficult to represent in floating point, while the initial conditions for *F*, the Heaviside step function, H(x), faces no such difficulties [24]. The Heaviside step function is defined to be equal to 0 for x<0 and to be equal to 1 for x≥0. At this point, we need to derive the PDE for the distribution *F*, starting from the PDE for the density, *f*, Eq. (5).

First, at the lower boundary, it is intuitive that the distribution should be zero, $F(x_{-},t)=0$, while f(1,t)=0 implies that at the upper boundary $\partial_{x}F(1,t)=0$. Inside the domain, the PDE itself reformulates as

$$\partial_{t}f(x,t)=\partial_{x}\left[\frac{1}{2}\partial_{x}\left[\beta^{2}f\right]-\left(\alpha -x+\gamma \sin\left(\Omega (t-\phi )\right)\right)f\right]$$

so that

$$\partial_{x}\partial_{t}F(x,t)=\partial_{x}\left[\frac{\beta^{2}}{2}\cdot \partial_{x}^{2}F-\left(\alpha -x+\gamma \sin\left(\Omega (t+\phi )\right)\right)\cdot \partial_{x}F\right].$$

Integrating with respect to *x* then gives

$$\partial_{t}F(x,t)=\frac{\beta^{2}}{2}\cdot \partial_{x}^{2}F-\left(\alpha -x+\gamma \sin\left(\Omega (t+\phi )\right)\right)\cdot \partial_{x}F+C(t),$$

where C(t) is a constant of integration depending on *t*. Now consider the lower boundary condition, $x=x_{-}$. Here, $F(x_{-},t)=0$ implies that $\partial_{t}F=0$ and so

(8) $$C(t)=-\left[\frac{\beta^{2}}{2}\cdot \partial_{x}^{2}F-\left(\alpha -x+\gamma \sin\left(\Omega (t+\phi )\right)\right)\cdot \partial_{x}F\right].$$

The right-hand side in Eq. (8) is precisely the reflecting boundary condition on *f* once we recall that $\partial_{x}F=f$. Therefore, C(t)≡0.

Thus, the fully specified PDE for *F*, which we will be solving frequently in what follows, is

(9) $$\begin{array}{rl} & \partial_{t}F^{\left(\phi \right)}(x,t)=\frac{\beta^{2}}{2}\cdot \partial_{x}^{2}F^{\left(\phi \right)}-\left(\alpha -x+\gamma \sin\left(\Omega (t+\phi )\right)\right)\cdot \partial_{x}F^{\left(\phi \right)},\\ & \left\{ \begin{array}{c} F^{\left(\phi \right)}(x,0)=H(x),\\ F^{\left(\phi \right)}(x,t){|}_{x=x_{-}}\equiv 0,\\ \partial_{x}F^{\left(\phi \right)}(x,t){|}_{x=1}\equiv 0. \end{array}\right. \end{array}$$

**Fig. 4:**
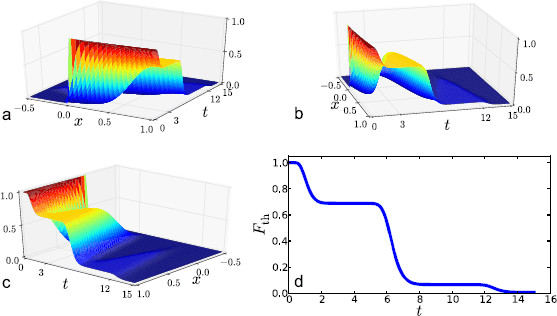
Example solution to Eq. (9) for $\left(\alpha ,\beta ,\gamma )=(0.5,0.3,0.5\sqrt{2}\right)$; Ω=1, ϕ=π/2. In **a**, **b**, **c**, we show the full solution in space–time F(x,t). In **d** we show the time solution at the upper boundary, F(1,t)

Numerical solutions for Eq. (9) are shown in Fig. 4. We have used the standard Crank–Nicholson finite-difference algorithm (central-differences in space with equally weighted implicit-explicit terms in time, see [25]). 

### 2.3 Fortet Equation

Consider a general form of Eq. (3),

$$dY_{t}=b(t,Y_{t})dt+\sigma (t,Y_{t})dW_{t}.$$

Let $\Phi (y,t|y_{0},t_{0}):=P[Y_{t}\leq y|Y_{t_{0}}=y_{0}]$ be the transition cumulative distribution of *Y*. Note that this is the distribution of $Y_{t}$ in absence of a threshold, different from the distribution given in Eq. (7), which is the distribution of the process constrained to be below the threshold. Now consider an arbitrary time-dependent threshold $v_{\mathrm{th}}(t)$. The Fortet equation (see [26]) convolves the first-hitting time probabilities, g(t), with the transition density of the process. Integrating over $\left(-\infty ,v_{\mathrm{th}}(t)\right)$, we obtain

(10) $$1-\Phi \left(v_{\mathrm{th}}(t),t|v_{0},0\right)=\int_{0}^{t}g(\tau )\left[1-\Phi \left(v_{\mathrm{th}}(t),t|v_{\mathrm{th}}(\tau ),\tau \right)\right]d\tau .$$

The left-hand side is simply the probability of exceeding $v_{\mathrm{th}}$ at time *t* starting at $v_{0}$ at time 0. This can also be written as the probability of hitting $v_{\mathrm{th}}$ for the first time at time τ<t and then exceeding $v_{\mathrm{th}}$ at time *t* starting at $v_{\mathrm{th}}$ at time *τ*, integrated over all *τ*.

The Fortet equation is particularly appealing to use when we have an analytical expression for *Φ*. For the problem at hand, *Φ* is complicated due to the time-dependent forcing. However, the following transformation yields a time-homogeneous *Y* for which *Φ* will be tractable, along with an associated moving threshold, $v_{\mathrm{th}}(t)$. This makes feasible the use of the Fortet equation. To obtain this transformation, cf. [27], consider the deterministic version of the SDE in Eq. (3)

(11) $$\begin{array}{rl} & dv(t)=\left(\alpha -v+\gamma \sin\left(\Omega (t+\phi )\right)\right)dt,\\ & v(0)=0 \end{array}$$

with solution

(12) $$\begin{array}{rl} v(t)= & \alpha \left(1-\exp(-t)\right)\\ & +\frac{\gamma }{\sqrt{1+\Omega^{2}}}\left[\sin\left(\Omega (t+\phi )-\psi \right)-\exp(-t)\sin(\phi \Omega -\psi )\right];\\ \psi = & \arctan(\Omega ). \end{array}$$

Now take $X_{t}$, the solution to Eq. (3) and v(t), Eq. (12), and let $Y_{t}=X_{t}-v(t)$. Then

(13) $$dY_{t}=-Y_{t}dt+\beta dW,$$

which has the time and parameter dependent threshold

(14) $${v_{\mathrm{th}}}_{\left\{\alpha ,\gamma ;\phi \right\}}(t)=v_{\mathrm{th}}-v(t).$$

That is, $X_{t}$ hits the constant threshold $v_{\mathrm{th}}$ if and only if $Y_{t}$ hits the moving threshold ${v_{\mathrm{th}}}_{\left\{\alpha ,\gamma ;\phi \right\}}(t)$, where the subindex indicates the dependence on *α*, *γ* and *ϕ*. Therefore, the ISIs produced by *X* and *Y* are the same and so are their distributions. Thus, $g_{\phi }(\tau )$ satisfies

(15) $$\begin{array}{rl} & 1-\Phi_{\left\{\beta \right\}}\left({v_{\mathrm{th}}}_{\left\{\alpha ,\gamma ;\phi \right\}}(t),t|0,0\right)\\ & \;=\int_{0}^{t}g_{\phi }(\tau )\left[1-\Phi_{\left\{\beta \right\}}\left({v_{\mathrm{th}}}_{\left\{\alpha ,\gamma ;\phi \right\}}(t),t|{v_{\mathrm{th}}}_{\left\{\alpha ,\gamma ;\phi \right\}}(\tau ),\tau \right)\right]d\tau , \end{array}$$

where

$$\Phi_{\left\{\beta \right\}}(y,t|y_{0},t_{0})=\frac{1}{\sqrt{\pi \beta^{2}(1-e^{-2(t-t_{0})})}}\int_{-\infty }^{y}\exp\left(-\frac{{\left(x-y_{0}e^{-(t-t_{0})}\right)}^{2}}{\beta^{2}(1-e^{-2(t-t_{0})})}\right)dx$$

is the conditional cumulative distribution function of $Y_{t}$ defined in Eq. (13).

## 3 Parameter Estimation Algorithms

The unknown parameters in Eq. (3) are *α*, *β*, and *γ*, while we assume *Ω* known. The reason why the amplitude, *γ*, is often unknown while the frequency, *Ω*, is known is that one can usually observe the sinusoidal input and thus its frequency. Further, the encoding of the input into neuronal firing patterns often involves phase locking to the sinusoidal component. However, the actual forcing amplitude at the level of the neuron is usually modified by various synaptic and other filtering processes, unless the cell receives direct sinusoidal current injection.

Our goal is to estimate the structural parameters (*α*, *β*, *γ*) from a sample of spike time data, $\left\{i_{1},\ldots ,i_{N}\right\}$. There are several algorithms for estimating the parameters for the simpler and more common case of γ=0. One such algorithm relies on the Fortet equation (see [7,8]), which we extend to the presence of a time-varying current. A more basic approach is to directly solve the Fokker–Planck equation for the probability density of $X_{t}$, [19-21], from which one can derive the survival distribution of $I_{n}$, and use this to compare against the empirical survival distribution of $I_{n}$ obtained from data. An approximate maximum likelihood approach is also possible by numerical differentiation. The relation between Fokker–Planck equations and the first-passage time problem is discussed in most introductory books on stochastic analysis; see, for example, [28]. A recent review of this approach for the simple γ=0 case in neuronal modeling can be found in [21], wherein the first passage problem is discussed at great lengths in the context of spiking neurons. We will use this in Sect. 2.2. A more elaborate approach using the Fokker–Planck equation to approximate the hitting time distribution is given in [29]. The techniques in [29] avoid the need to compute the Fokker–Planck PDE numerically, instead approximating it with analytically known solutions. This approach might offer significant computational savings, but since this would at most amount to a computational speed-up of our algorithm, we have left this unexplored for now. 

The immediate problem in generalizing the aforementioned approaches to the case of γ≠0 is that the $I_{n}$’s are no longer identically distributed since the phase $\phi_{n-1}$ of the *n*th interval $I_{n}$ depends on $t_{n-1}$, the time the previous spike occurred. The $I_{n}$’s are also dependent, but conditionally independent given $\phi_{n-1}$. So the trajectories in each interval are parameterized by the value of $\phi_{n-1}$ at the time of the last spike/reset. We overcome this obstacle by splitting the $I_{n}$’s in groups, and approximating the $I_{n}$’s within groups as coming from identically distributed trajectories in a sense to be specified below. This approximation which solves the challenge of dependent and non-identically distributed ISIs is the primary contribution of this paper.

### 3.1 *ϕ*-Binning

Before we can use Eq. (9) or (15), we need to deal with the fact that *ϕ* is not fixed, but instead each $I_{n}$ starts with a distinct $\phi_{n}$. Our approach is to partition the interval [0,2π/Ω] into *M* bins, where M≪N, and represent each bin by the midpoint of the bin, $\phi_{m}$. Then we approximate the *N* observed $\phi_{n}$’s by the closest $\phi_{m}$ and pretend that any observed $I_{n}$ was not produced by a trajectory of the form in Eq. (3) with $\phi =\phi_{n}$, but with $\phi =\phi_{m}$. Our hope is that for a judicious choice of *M*, we can balance the error of $\phi_{n}\neq \phi_{m}$ with having enough data points in each bin in order to obtain a useful estimate from Eq. (9) or (15).

There is clearly much freedom in how one sets up these bins, but we will do the simplest thing and make them all of equal width, δϕ=2π/(ΩM). Each $\phi_{n}$ will belong to one and only one of the bins ${\left[\phi_{m}-\delta \phi /2,\phi_{m}+\delta \phi /2\right)}_{m=1}^{M}$, with center points $\phi_{m}=\delta \phi /2+(m-1)\delta \phi$, for m=1,…,M. Thus, given an empirically observed $I_{n}$ with associated $\phi_{n}$, we will pretend that it was produced by the process

$$dX_{s}=(\alpha -X_{s})ds+\gamma \sin\left(\Omega \left(s+\phi_{m}(n)\right)\right)ds+\beta dW_{s},$$

where

$$\phi_{m}(n)=\underset{\phi_{m}}{arg min}|\phi_{n}-\phi_{m}|.$$

**Fig. 5:**
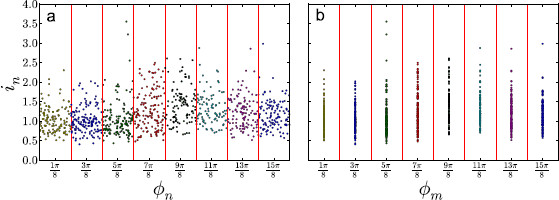
The raw $\left(i_{n},\phi_{n}\right)$ pairs (**a**) are binned into a set of *M* bins with a representative $\phi_{m}$ (**b**) and the ISIs within each bin are treated as a renewal process. In this illustration, M=8, Ω=1 while the parameters *α*, *β*, *γ* are taken from the supra-threshold regime

This binning is illustrated in Fig. 5. 

**Fig. 6:**
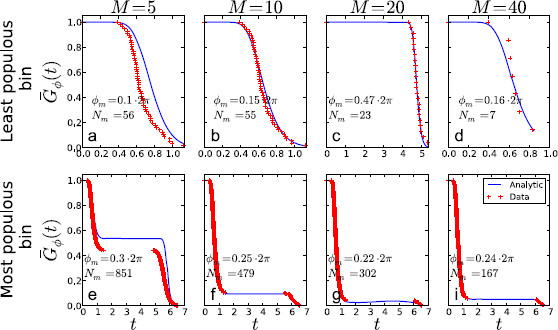
Effect of *M*, the number of bins, on the approximate survival distribution. *The full-drawn blue curve* is the true survivor distribution given in Eq. (9), *the red points* are the approximation given in Eq. (16). In the figures, the least populous (*above*) and most populous (*below*) bin for each *M* is shown. The width of the bins is δϕ=2π/(ΩM). We have used **a**, **e**M=5; **b**, **f**M=10; **c**, **g**M=20; **d**, **i**M=40. As *M* increases, the approximation of using the survival distribution using $\phi_{m}$ instead of $\phi_{n}$ improves since $\phi_{m}(n)\to \phi_{n}$ as M→∞. The data are generated using parameter values from the supersinusoidal regime and N=1000. For this particular data set the largest generated ISI was 6.55 time units

While we have no rigorous approach to determine the value of *M*, our limited experience suggests that given N=1000 ISIs, M=10, or M=20 gives satisfactory results for very different parameter regimes. In general, choosing *M* is a balancing act. For *M* too high, the resulting bins will have too few data points to approximate $\bar{G}(I)$ accurately. Therefore, *M* is forced to be small when sample size is not large. For *M* too low, the approximation of the phase shifts will be poor, leading to a biased estimate of $\bar{G}(I)$. We illustrate the effect of increasing *M* in Fig. 6. Generally, as long as there are sufficient data points, as *M* increases, the approximation of using the survival distribution with $\phi_{m}$ instead of $\phi_{n}$ improves since $\phi_{m}(n)\to \phi_{n}$ as M→∞. In the sequel, we will use M=20 for sample sizes of N=1000 and M=8 for sample sizes of N=100. 

### 3.2 Fokker–Planck Algorithm

Within each bin it is clear how to apply Eq. (7). In the *m*th bin, for a given $\phi_{m}$, we approximate ${\bar{G}}_{\phi }(t)$ by

(16) $${\hat{\bar{G}}}_{\phi_{m}}(t)=\frac{\#[i_{n}>t|\phi_{n-1}\in [\phi_{m}-\delta \phi /2,\phi_{m}+\delta \phi /2)]}{N_{m}},$$

where $N_{m}$ is the number of ISIs in bin *m*. Using Eq. (7), we define the loss function

(17) $$L(\alpha ,\beta ,\gamma )=\sum_{\phi_{m}}N_{m}\left\{\underset{t>0}{\sup}|{\hat{\bar{G}}}_{\phi_{m}}(t)-F_{\alpha ,\beta ,\gamma }^{\phi_{m}}(x_{\mathrm{th}},t)|\right\}.$$

The weight $N_{m}$ is included so that bins with larger sample sizes have a larger influence on the estimates.

To evaluate the supremum in Eq. (17), we spline interpolate the empirically discrete $\hat{\bar{G}}$ for each $\phi_{m}$, sample at the time nodes of the PDE discretization and finally take the maximum amongst the sampled values. We then minimize *L* using an optimization algorithm (see below, Sect. 4) and take our estimates $\hat{\alpha }$, $\hat{\beta }$, $\hat{\gamma }$ to be

$$\hat{\alpha },\hat{\beta },\hat{\gamma }=\underset{\alpha ,\beta ,\gamma }{arg min}L(\alpha ,\beta ,\gamma ).$$

Note that the relation between the spike time survival density, ${\bar{G}}_{\phi }$ and the transition distribution, $F_{\phi }$, in Eq. (7) could also allow for an approximate maximum likelihood estimator (MLE), based on maximizing

$$L^{\mathrm{MLE}}(\alpha ,\beta ,\gamma )=\sum_{n}\log\left(g_{\phi_{n-1}}(i_{n})\right)=\sum_{n}\log\left[-\partial_{t}F_{\alpha ,\beta ,\gamma }^{\phi_{n-1}}(x_{\mathrm{th}},t)\right]{|}_{t=i_{n}},$$

where the derivative has to be approximated by finite differences. We can then again use binning to avoid having to compute the PDE separately for each $\left(i_{n},\phi_{n-1}\right)$. Our experience with the MLE approach has been that the quality of the estimates provided are similar to those obtained by minimizing Eq. (17) and that the associated computing times are on the same order. Due to this similarity and in order to keep the paper concise, we include details of the MLE estimates only in the supplementary online material.

### 3.3 Fortet Algorithm

An alternative approach is to form a loss function from Eq. (15). This is similar to what is done in [7,8] for the simpler case of a constant threshold. Noting that $\int_{0}^{t}g(\tau )[1-\Phi ]d\tau =E[(1-\Phi )1_{I\leq t}]$ where the expectation is taken with respect to the distribution of the random variable *I*, we can use the fact that the ISIs are approximately independent and invoke the law of large numbers to estimate the integral as

$$\begin{array}{rl} & \int_{0}^{t}g_{\phi_{m}}(\tau )\left[1-\Phi_{\left\{\beta \right\}}^{\left(\phi \right)}\left({v_{\mathrm{th}}}_{\left\{\alpha ,\gamma ;\phi \right\}}(t),t|{v_{\mathrm{th}}}_{\left\{\alpha ,\gamma ;\phi \right\}}(\tau ),\tau \right)\right]d\tau \\ & \;\approx \frac{1}{N_{m}}\sum_{i_{n}<t}\left[1-\Phi_{\left\{\beta \right\}}^{\left(\phi \right)}\left({v_{\mathrm{th}}}_{\left\{\alpha ,\gamma ;\phi \right\}}(t),t|{v_{\mathrm{th}}}_{\left\{\alpha ,\gamma ;\phi \right\}}(i_{n}),i_{n}\right)\right]. \end{array}$$

We then define the loss function

(18) $$\begin{array}{rl} L(\alpha ,\beta ,\gamma )= & \sum_{\phi_{m}}N_{m}\{\underset{s>0}{\sup}|1-\Phi_{\left\{\beta \right\}}^{\left(\phi_{m}\right)}\left({v_{\mathrm{th}}}_{\left\{\alpha ,\gamma ;\phi \right\}}(s),s|0,0\right)\\ & -\frac{1}{N_{m}}\sum_{i_{n}<s}\left[1-\Phi_{\left\{\beta \right\}}^{\left(\phi_{m}\right)}\left({v_{\mathrm{th}}}_{\left\{\alpha ,\gamma ;\phi \right\}}(s),s|{v_{\mathrm{th}}}_{\left\{\alpha ,\gamma ;\phi \right\}}(i_{n}),i_{n}\right)\right]|\\ & /\omega (\phi_{m};\alpha ,\beta ,\gamma )\}. \end{array}$$

We divide each inner term by $\omega (\phi_{m};\alpha ,\beta ,\gamma )=\sup_{s>0}|1-\Phi_{\alpha ,\beta ,\gamma }^{\left(\phi_{m}\right)}(v_{\mathrm{th}}(s),s|v_{0})|$, following the suggestion in [8]. This scaling ensures that Eq. (15) divided by ω(α,β,γ) will vary between 0 and 1 for all parameter values thus giving sense to the measure defined by the loss function. Since we can solve in closed form for *Φ*, we have all we need given an observed spike train of $i_{n}$’s. We evaluate the sup by sampling at K=500 uniformly spaced points in $\left(0,I_{\max}+\epsilon \right]$ and taking the maximum of the sampled values.

### 3.4 Initialization of the Algorithms

The parameter search can be initialized in a simple way using the fact that the Fokker–Planck PDE is almost an “advection-diffusion” equation whose solution is almost a Gaussian. Then $\bar{G}(t)$ can be approximated by the amount of probability mass of a Gaussian to the left of the threshold at time *t*. The idea is as follows. Suppose we are solving the following PDE:

(19) $$\partial_{t}\rho =-U\partial_{x}[\rho ]+\frac{\beta^{2}}{2}\partial_{x}^{2}[\rho ].$$

Its solution given an initial condition ρ(x,0)=δ(x) will be a Gaussian bell moving to the right with speed *U* and standard deviation $\sigma =\beta \sqrt{t}$.

The survivor function $\bar{G}(t)$ can be thought of as the amount of area that has passed the threshold (from the left moving to the right). We can then invert the information about $\bar{G}$ to estimate *U* and *β*. In particular, a Gaussian bell has ≈0.158 of its mass more than one standard deviation to the right of its mean. Thus, at time $t_{1}$ such that $\bar{G}(t_{1})=0.842$, the right tail of more than one standard deviation of the Gaussian bell has crossed the threshold. The threshold is at x=1 and we obtain the following equation:

(20) $$Ut_{1}+\beta \sqrt{t_{1}}=1.$$

Similarly, at time $t_{2}$ such that $\bar{G}(t_{2})=1-0.842$, the Gaussian bell has crossed the threshold except for the left tail and we have

(21) $$Ut_{2}-\beta \sqrt{t_{2}}=1.$$

If *U* and *β* were constant, then Eqs. (20) and (21) provide two equations in two unknowns. However, U=U(x,t)=(α−x+γsin(Ω(t+ϕ))) is not constant and we approximate *U* as

(22) $$U(x,t)\approx \alpha -0.5+\gamma \frac{1}{t}\int_{0}^{t}\sin\left(\Omega (\tau +\phi )\right)d\tau ,$$

i.e., we approximate the space-dependent term, *x*, with the mid-point between the reset value, $v_{0}=0$, and the threshold, $v_{\mathrm{th}}=1$, and we approximate the time-dependent term, sin(Ω(τ+ϕ)), by its time-average value between 0 and *t*. If we use the 0th, 1st, and 2nd standard deviation points, we can form 5 equations in 3 unknowns as follows:

$$\begin{array}{rcl} \alpha t_{1}+\gamma s(t_{1})+2\beta \sqrt{t_{1}} & = & 1+0.5t_{1},\\ \alpha t_{2}+\gamma s(t_{2})+\beta \sqrt{t_{2}} & = & 1+0.5t_{2},\\ \alpha t_{3}+\gamma s(t_{3})+0\beta & = & 1+0.5t_{3},\\ \alpha t_{4}+\gamma s(t_{4})-1\beta \sqrt{t_{4}} & = & 1+0.5t_{4},\\ \alpha t_{5}+\gamma s(t_{5})-2\beta \sqrt{t_{5}} & = & 1+0.5t_{5} \end{array}$$

with the time-average weighting function s(t)=(cos(Ωϕ)−cos(Ω(t+ϕ)))/Ω. However, the approximation is best for earlier times, when the solution is closer to a Gaussian bell that is approaching the threshold, but less correct for later times, since it neglects the loss of probability mass and thus overestimates the backward probability current. Indeed, we have found it to be best to use only $t_{1}$ and $t_{2}$. In the following we use only these equations:

$$\begin{array}{rcl} \alpha t_{1}+\gamma s(t_{1})+2\beta \sqrt{t_{1}} & = & 1+0.5t_{1},\\ \alpha t_{2}+\gamma s(t_{2})+\beta \sqrt{t_{2}} & = & 1+0.5t_{2} \end{array}$$

for the initializer. We can form these equations separately for each $\phi_{m}$ bin, thus resulting in M×2 equations for the unknowns *α*, *β*, and *γ*. Since we have more equations than unknowns, we use least-squares estimates in a regression to pick out unique *α*, *β*, and *γ* estimates.

**Fig. 7:**
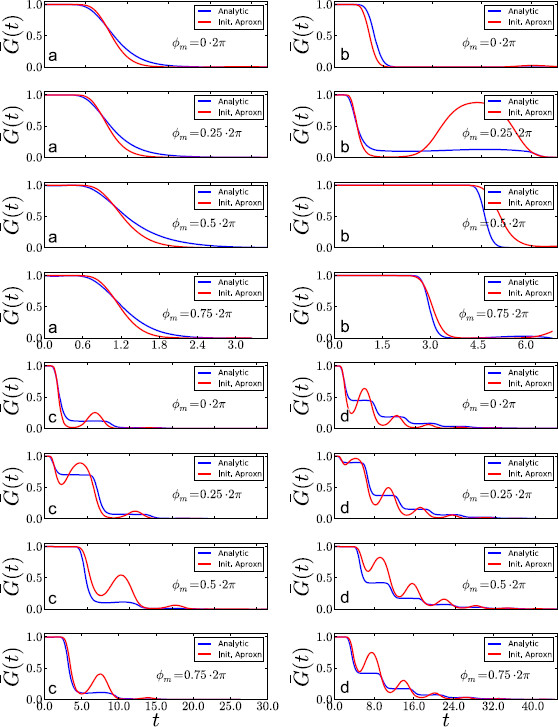
*The blue curves* are the numerically obtained survivor distributions ${\bar{G}}_{\phi }$ for the exact parameters in the four regimes (as in Table 1) and Ω=1. *The red curves* are obtained in the following manner: Simulations using the true parameters were used to generate sample spikes. Using these samples, the initializer algorithm was used to generate estimates for *α*, *β*, *γ*. Using these estimates, the bell curve discussed in Sect. 3.4 was formed and evolved in time. Thus, *the red curve* drawn in the figures measures the area under this bell that is to the left of the threshold at time *t*. **a** supra-threshold, **b** supersinusoidal, **c** critical, **d** subthreshold

The proposed initialization procedure has two advantages. First, it is automatic, i.e., it requires only the data and no input or guidance from the user. Second, it is extremely fast. While the precise effect of the initializer is shown in Sect. 4, it is intuitively clear that it will work best in the supra-threshold parameter regime when the bell curve is truly moving past the threshold as a whole and less so for subthreshold regimes, when only the diffusive force serves to propel the process to reach $v_{\mathrm{th}}$. The behavior of the initializer in the different regimes is illustrated in Fig. 7. What we show in Fig. 7 is the following: First, we show the survival distribution for a given regime and $\phi_{m}$ fixed. Then using data generated from such a regime and with $\phi_{n}$ in the *m*th bin, the initializer tries to find the best approximation by the motion of a Gaussian bell which will fit these data, in the sense of solving for *α*, *β*, *γ* as previously described. Once this is done, we then show in red the amount of area under this Gaussian bell to the left of the threshold. Of course, the interpretation of the survival distribution for an ISI as a fraction of the area under a moving bell with conserved total area is wrong, but the assumption is useful in automatically generating initial values for the more appropriate approximations to start their work. 

## 4 Method Comparison on Simulated Data

We will now use our algorithms on spike trains simulated from the four different regimes: the supra-threshold, the critical, the supersinusoidal and the subthreshold. We have used 100 sample spike trains per regime, with N=100 as well as N=1000 spikes per train. In order to perform the numerical minimization of Eqs. (17) and (18), we have used an implementation of the Nelder–Mead algorithm from the SciPy library [30]. The Nelder–Mead algorithm is a non-linear minimization routine which uses a bounding-polygon method to zero-in on the minimum and thus avoids the need to provide the gradient of the loss function. It is the standard non-gradient minimization algorithm. 

**Fig. 8:**
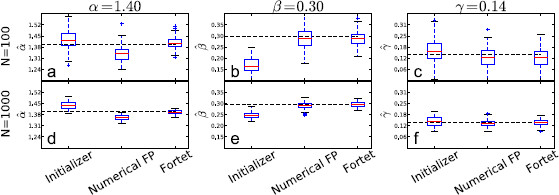
Boxplots of parameter estimates for the supra-threshold regime. *The upper plots* (**a**, **b**, **c**) show estimates using N=100 sample spikes per estimation, while *the lower plots* (**d**, **e**, **f**) use N=1000. *The dashed line* indicates the true parameter value, while *the red line inside the boxes* indicates the median of the estimates. *The boxes* contain the central 50 % of the estimates. *The bars* indicate the range of the estimates, except for outliers given by *the points outside the bars*, and defined to be more than 1.5 times the interquantile range (the height of *the box*) from *the box*

**Fig. 9:**
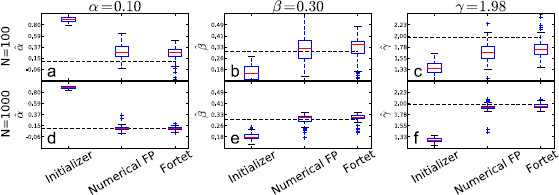
Boxplots of parameter estimates for the supersinusoidal regime. *The upper plots* (**a**, **b**, **c**) show estimates using N=100 sample spikes per estimation, while *the lower plots* (**d**, **e**, **f**) use N=1000. *The dashed line* indicates the true parameter value, while *the red line inside the boxes* indicates the median of the estimates. *The boxes* contain the central 50 % of the estimates. *The bars* indicate the range of the estimates, except for outliers given by *the points outside the bars*, and defined to be more than 1.5 times the interquantile range (the height of *the box*) from *the box*

**Fig. 10:**
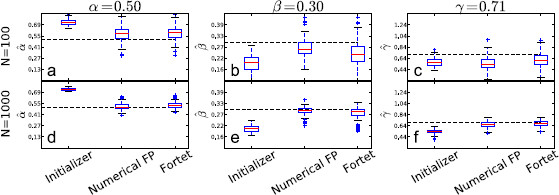
Boxplots of parameter estimates for the critical regime. *The upper plots* (**a**, **b**, **c**) show estimates using N=100 sample spikes per estimation, while *the lower plots* (**d**, **e**, **f**) use N=1000. *The dashed line* indicates the true parameter value, while *the red line inside the boxes* indicates the median of the estimates. *The boxes* contain the central 50 % of the estimates. *The bars* indicate the range of the estimates, except for outliers given by *the points outside the bars*, and defined to be more than 1.5 times the interquantile range (the height of *the box*) from *the box*

**Fig. 11:**
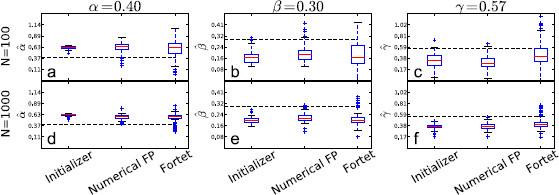
Boxplots of parameter estimates for the subthreshold regime. *The upper plots* (**a**, **b**, **c**) show estimates using N=100 sample spikes per estimation, while *the lower plots* (**d**, **e**, **f**) use N=1000. *The dashed line* indicates the true parameter value, while the red line inside the boxes indicates the median of the estimates. *The boxes* contain the central 50 % of the estimates. *The bars* indicate the range of the estimates, except for outliers given by *the points outside the bars*, and defined to be more than 1.5 times the interquantile range (the height of *the box*) from *the box*

The estimation results are shown in Figs. 8, 9, 10, 11, where we plot box plots for the estimates, $\hat{\alpha }$, $\hat{\beta }$, $\hat{\gamma }$ in the four regimes. We also tabulate the average and the empirical 95 % confidence intervals of the estimates in Tables 2 and 3. Conclusions that can be drawn from these results are as follows. The initializer method is effective for the supra-threshold regime and gives reasonable ballpark estimates for all regimes, though the error can be substantial for the supersinusoidal regime. In general, both the Fortet and Fokker–Planck algorithm estimate the parameters well in the supra-threshold, critical and supersinusoidal regimes. The estimators’ variance is especially low in the supra-threshold regime, while it is higher for the critical and supersinusoidal regimes. In the supersinusoidal regime, the two algorithms give accurate estimates even though the initializer can be quite off. On the other hand, in the subthreshold regime, the initializer has a performance comparable to that of the two more involved methods. It seems that distinguishing between the constant bias and the sinusoidal current is difficult if their sum is not sufficient to generate spikes without noise. 

**Table 2:** Averages and empirical 95 % confidence intervals of the estimates for N=100 spikes per train

Parameter	Initializer	Fokker–Planck	Fortet
Supra-threshold regime			
*α* = 1.40	1.43: [1.29,1.56]	1.34: [1.24,1.43]	1.41: [1.33,1.49]
*β* = 0.30	0.17: [0.10,0.24]	0.29: [0.21,0.39]	0.29: [0.22,0.36]
*γ* = 0.14	0.16: [0.02,0.33]	0.12: [0.02,0.23]	0.12: [0.01,0.24]
Supersinusoidal regime			
*α* = 0.10	0.92: [0.83,1.01]	0.28: [0.02,0.59]	0.24: [−0.22,0.42]
*β* = 0.30	0.15: [0.10,0.25]	0.31: [0.14,0.53]	0.32: [0.14,0.46]
*γ* = 1.98	1.35: [1.13,1.57]	1.67: [1.33,2.05]	1.77: [1.44,2.38]
Critical regime			
*α* = 0.50	0.72: [0.66,0.80]	0.57: [0.32,0.73]	0.57: [0.36,0.73]
*β* = 0.30	0.19: [0.10,0.26]	0.27: [0.17,0.40]	0.25: [0.15,0.40]
*γ* = 0.71	0.57: [0.44,0.73]	0.55: [0.30,0.83]	0.62: [0.38,0.93]
Subthreshold regime			
*α* = 0.40	0.62: [0.57,0.67]	0.63: [0.33,0.84]	0.58: [0.03,1.00]
*β* = 0.30	0.17: [0.10,0.29]	0.20: [0.10,0.37]	0.19: [0.00,0.41]
*γ* = 0.57	0.32: [0.00,0.53]	0.29: [0.00,0.62]	0.46: [0.00,1.19]

**Table 3:** Averages and empirical 95 % confidence intervals of the estimates for N=1000 spikes per train

Parameter	Initializer	Fokker–Planck	Fortet
Supra-threshold regime			
*α* = 1.40	1.44: [1.40,1.50]	1.36: [1.33,1.40]	1.40: [1.37,1.42]
*β* = 0.30	0.25: [0.22,0.28]	0.29: [0.26,0.32]	0.30: [0.27,0.32]
*γ* = 0.14	0.14: [0.10,0.19]	0.14: [0.10,0.17]	0.14: [0.10,0.18]
Supersinusoidal regime			
*α* = 0.10	0.90: [0.85,0.92]	0.11: [0.03,0.29]	0.10: [0.03,0.16]
*β* = 0.30	0.18: [0.14,0.23]	0.30: [0.21,0.34]	0.31: [0.22,0.34]
*γ* = 1.98	1.26: [1.16,1.34]	1.92: [1.49,2.05]	1.96: [1.86,2.07]
Critical regime			
*α* = 0.50	0.73: [0.70,0.75]	0.51: [0.43,0.63]	0.53: [0.45,0.64]
*β* = 0.30	0.20: [0.17,0.24]	0.29: [0.24,0.32]	0.28: [0.19,0.33]
*γ* = 0.71	0.54: [0.44,0.61]	0.66: [0.52,0.76]	0.67: [0.54,0.77]
Subthreshold regime			
*α* = 0.40	0.62: [0.55,0.65]	0.57: [0.45,0.66]	0.56: [0.26,0.71]
*β* = 0.30	0.20: [0.17,0.26]	0.22: [0.18,0.29]	0.21: [0.13,0.35]
*γ* = 0.57	0.36: [0.18,0.44]	0.36: [0.25,0.50]	0.43: [0.28,0.72]

**Fig. 12:**
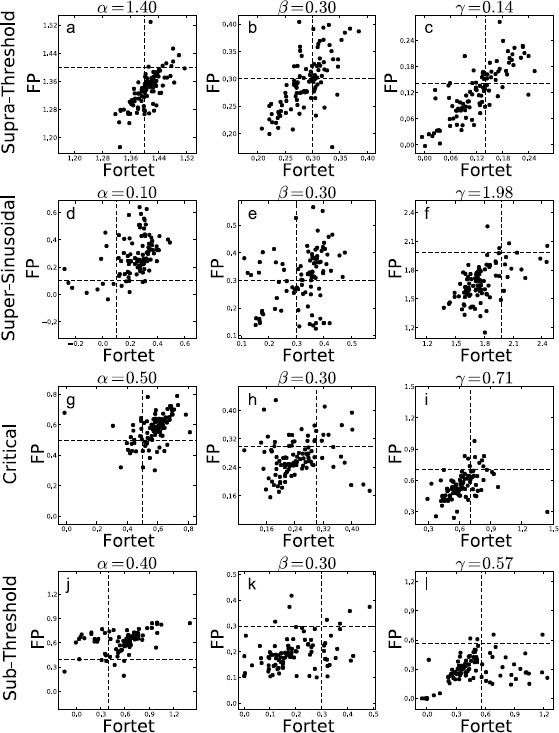
Estimates based on samples of N=100 spikes obtained from the Fokker–Planck algorithm against the Fortet algorithm for the four different parameter regimes, with parameter values given in Table 1, fixing Ω=1. *Each row* corresponds to one regime and one set of simulations. *Each column* corresponds to a parameter, with the specific value indicated *above each plot*. **a**, **b**, **c** supra-threshold; **d**, **e**, **f** supersinusoidal; **g**, **h**, **i** critical; **j**, **k**, **l** subthreshold

**Fig. 13:**
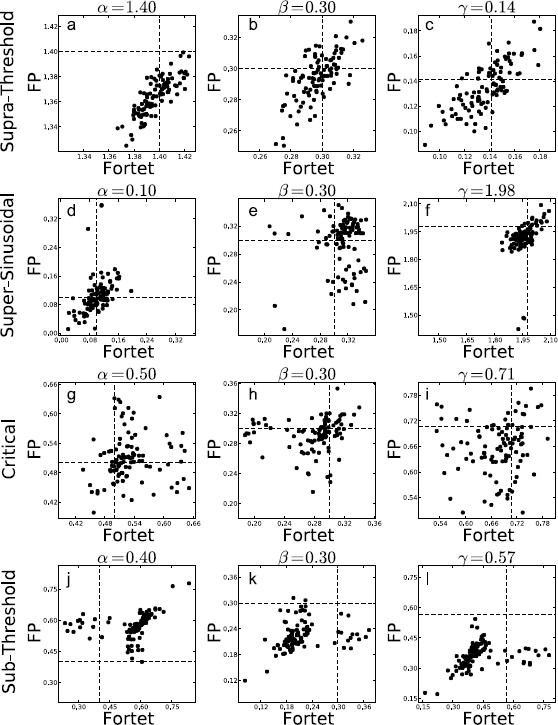
Estimates based on samples of N=1000 spikes obtained from the Fokker–Planck algorithm against the Fortet algorithm for the four different parameter regimes, with parameter values given in Table 1, fixing Ω=1. *Each row* corresponds to one regime and one set of simulations. *Each column* corresponds to a parameter, with the specific value indicated *above each plot*. **a**, **b**, **c** supra-threshold; **d**, **e**, **f** supersinusoidal; **g**, **h**, **i** critical; **j**, **k**, **l** subthreshold

The Fokker–Planck method has a larger bias but a smaller spread than the Fortet method for N=100, Table 2. However for N=1000, the two methods have comparable spreads, while the Fortet method retains a smaller bias, see Table 3. More precisely, for N=1000, the Fokker–Planck method has a smaller spread in the subthreshold regime, while the Fortet method has a smaller spread in the supersinusoidal regime. As such, at least in the supersinusoidal regime, the Fortet method seems superior. A detailed comparison between the Fortet and the Fokker–Planck estimators for each parameter in each regime can be seen in Fig. 12 for N=100 and in Fig. 13 for N=1000. 

The two algorithms are numerically intensive. For N=100 and N=1000 spikes, we show the times taken for the estimation in Table 4. While we have done most of our numerical work in Python/SciPy [30], we have implemented the critical components of both algorithms in C. That is, we solve the inner part of Eq. (18) and the Fokker–Planck PDE, Eq. (9), in C using the GSL libraries [31]. From Table 4, we can verify that the computing time for the Fortet algorithm scales proportionally with the number of spikes. This is to be expected, since the Fortet equation has a term of the form $\sum_{i_{n}}$ which in turn has *N* terms and this forms the bulk of the computing time for the Fortet equation. The Fokker–Planck algorithm, on the other hand, scales less-than-linearly with *N*, since the dependency on *N* is in forming the approximation, $\hat{\bar{G}}$ to the survivor function and that is not computationally intensive (solving the PDE is). 

**Table 4:** Average times ± standard deviations in seconds for the algorithm in various regimes. *Left*: N=100 spikes; *right*: N=1000 spikes

Regime	Fortet	Fokker–Planck
Subthreshold	1.29 ± 0.72	0.52 ± 0.21
Supra-threshold	0.83 ± 0.28	0.18 ± 0.20
Critical	0.94 ± 0.42	0.36 ± 0.16
Supersinusoidal	1.36 ± 0.46	0.43 ± 0.17
Subthreshold	9.68 ± 4.98	1.69 ± 0.91
Supra-threshold	3.90 ± 1.05	0.21 ± 0.06
Critical	10.03 ± 2.88	1.28 ± 0.41
Supersinusoidal	10.13 ± 2.24	1.06 ± 0.33

## 5 The Effect of *Ω*

So far, we have held *Ω* constant and equal to 1. We now investigate the effect of varying *Ω* on the quality of estimates. To narrow the scope, we focus on increasing *Ω* while keeping the parameters in the critical regime such that $\alpha +\gamma /\sqrt{1+\Omega^{2}}=1$ and α=0.5. This amounts to increasing *γ* with *Ω*. We do the estimations for four values of Ω=[1,5,10,20]. Similarly to the previous section, we use 100 sample spike trains per parameter set, with each spike train consisting of N=1000 ISIs.

**Fig. 14:**
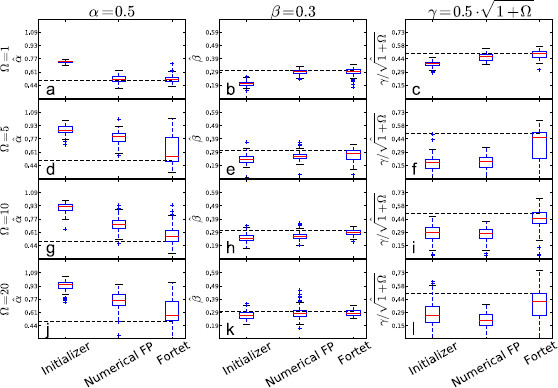
Boxplots of parameter estimates for varying *Ω* across [1,5,10,20] while holding $\gamma /\sqrt{1+\Omega^{2}}$ constant as to keep the parameters in the critical regime. **a**–**c**Ω=1, **d**–**f**Ω=5, **g**–**i**Ω=10, **j**–**l**Ω=20. *The boxes* contain the central 50 % of the estimates. *The bars* indicate the range of the estimates, except for outliers given by *the points outside the bars*, and defined to be more than 1.5 times the interquantile range (the height of *the box*) from *the box*

**Fig. 15:**
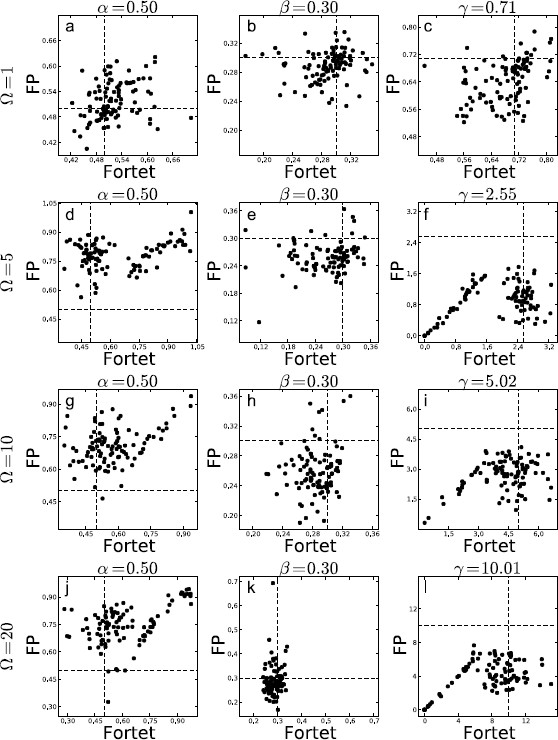
Estimates based on samples of N=1000 spikes obtained from the Fokker–Planck algorithm against the Fortet algorithm for a parameter set in the critical regime, while varying *Ω* across [1,5,10,20] and holding $\gamma /\sqrt{1+\Omega^{2}}$ and *α* constant. **a**, **b**, **c**Ω=1; **d**, **e**, **f**Ω=5; **g**, **h**, **i**Ω=10; **j**, **k**, **l**Ω=20

**Fig. 16:**
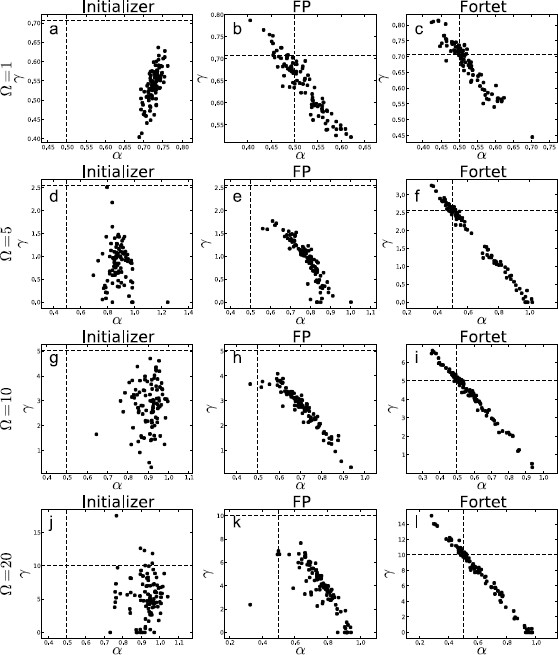
Comparison of $\hat{\alpha }$ vs. $\hat{\gamma }$ parameter estimates while varying *Ω* across [1,5,10,20], holding $\gamma /\sqrt{1+\Omega^{2}}$ constant as to keep the parameters in the critical regime. **a**, **b**, **c**Ω=1; **d**, **e**, **f**Ω=5; **g**, **h**, **i**Ω=10; **j**, **k**, **l**Ω=20

We show box plots of the estimates for each *Ω* in Fig. 14. We then directly compare the two algorithms, Fortet vs. Fokker–Planck, in Fig. 15. The immediate observation is that the Fokker–Planck algorithm fails to keep up at the higher frequencies and consistently underestimates *γ*. The Fortet algorithm does better, but still underestimates *γ*. In general, this underestimation of *γ* is accompanied by an overestimation of *α*. This is exacerbated at higher *Ω*. We illustrate the relation between estimates for *α* vs. *γ* in Fig. 16, where it is quite clear that an underestimation of *γ* is proportional to the overestimation of *α*. 

For completeness, we also include the estimates’ average and empirical 95 % confidence intervals in Table 5. 

**Table 5:** Averages and empirical 95 % confidence intervals of estimates for N=1000 spikes per train in the critical regime for varying *Ω* across [1,5,10,20]. Note that the upper subtable corresponds to the third subtable in Table 3; numbers differ slightly due to statistical fluctuations in the simulations

Parameter	Initializer	Fokker–Planck	Fortet
*Ω* = 1			
*α* = 0.50	0.73: [0.69,0.75]	0.52: [0.45,0.61]	0.52: [0.44,0.62]
*β* = 0.30	0.20: [0.17,0.25]	0.29: [0.24,0.33]	0.29: [0.22,0.34]
*γ* = 0.71	0.54: [0.44,0.62]	0.64: [0.53,0.75]	0.68: [0.55,0.81]
*Ω* = 5			
*α* = 0.50	0.88: [0.76,0.99]	0.78: [0.61,0.89]	0.64: [0.39,0.99]
*β* = 0.30	0.24: [0.17,0.31]	0.26: [0.20,0.34]	0.27: [0.12,0.34]
*γ* = 2.55	0.85: [0.00,1.65]	0.92: [0.00,1.68]	1.86: [0.00,3.10]
*Ω* = 10			
*α* = 0.50	0.90: [0.78,0.99]	0.71: [0.52,0.88]	0.58: [0.37,0.86]
*β* = 0.30	0.25: [0.18,0.33]	0.26: [0.20,0.35]	0.28: [0.23,0.32]
*γ* = 5.02	2.82: [0.92,4.38]	2.72: [0.95,3.88]	4.32: [1.20,6.49]
*Ω* = 20			
*α* = 0.50	0.93: [0.76,1.02]	0.75: [0.50,0.92]	0.62: [0.31,0.97]
*β* = 0.30	0.27: [0.20,0.33]	0.29: [0.20,0.43]	0.29: [0.25,0.33]
*γ* = 10.01	5.35: [0.00,12.29]	3.98: [0.00,6.83]	7.48: [0.00,13.96]

## 6 Discussion and Outlook

We have shown two methods to estimate parameters in Eq. (2) from ISI data. Our methods are based on binning the spikes into bins with representative phase shifts. We have devised a constructive procedure to automatically initialize the methods from the data.

Our computational results suggest that for low frequencies the Fortet algorithm is superior for large sample sizes, especially in the supersinusoidal regime, while the Fokker–Planck algorithm has a comparable accuracy and a lower variance for small sample sizes. Both algorithms find sensible estimates most of the time, although they seem less effective in the subthreshold regime. Their performance can be partially attributed to the ability of the initializer algorithm to supply good guesses for starting the optimization iterations.

The Fokker–Planck equation allows for approximate maximum likelihood estimation. We chose an alternative loss function, though, because it marginally appeared more robust, possibly because a numerical derivation step is avoided. This is further investigated by simulations in the supplementary online material. The simulations suggest that the distribution of the maximum likelihood estimates in the supersinusoidal regime appears bimodal, which is not the case for the alternative loss function, Eq. (17).

We have also made a preliminary exploration of the effect of *Ω* on the quality of the estimates. Our results show that an increase in *Ω* makes the parameters *α* and *γ* more difficult to estimate accurately and at high *Ω*, *γ* is underestimated, while *α* is over-estimated. We find that in this scenario, the Fortet algorithm does a markedly more accurate job then the Fokker–Planck algorithm.

We have assumed the time-constant *τ* of the leak term to be known. In most experiments that is not realistic, and it would be preferable to estimate *τ* alongside the other parameters. However, it is difficult to estimate [32]. When we tried to estimate it together with the other parameters, we usually obtained results which were not accurate. The obtained estimates resulted in ISIs that very well matched the data, no worse than the ISIs obtained from the true parameters. This leads us to believe that the simultaneous estimation of *τ* along with *α*, *β*, *γ* using only ISI data suffers from identifiability problems. In [5], they were able to estimate *τ* in the simpler nonsinusoidally-driven model, but concluded that adding *τ* as an unknown dramatically reduced the accuracy in the estimation of the other unknown parameters. The reason is that if *τ* is also estimated from a single dataset alongside the other parameters, then a reasonable fit can be found to the data for various combinations of *α*, *β*, *γ*, and *τ*, but the so-obtained parameter values can be far from the true values.

Our model is relatively simple and ignores neurophysiological realism, such as the fact that the spiking threshold is likely nonconstant, with a time-dependent functional form that would involve further unknown parameters. A recent paper attempting the parameter estimation in such a model, but without sinusoidal forcing, is [20]. Furthermore, intracellular recordings suggest that a hard threshold is a rough approximation and an exponential voltage-dependent spiking intensity is more realistic [33]. 

While our work has used a very specific form of the periodic forcing term, namely γsin(Ωt), it is clear how to apply the approach to an arbitrary periodic function. This can be done as long as one knows where in the period of oscillation a spike has occurred. If that is the case, then the binning procedure can be applied and the estimation methods proposed can be attempted.

## Competing Interests

The authors declare that they have no competing interests.

## Authors’ Contributions

All authors contributed equally to the writing of this paper. All authors read and approved the final manuscript.
